# The first report of *Cryptosporidium andersoni* in horses with diarrhea and multilocus subtype analysis

**DOI:** 10.1186/s13071-015-1102-0

**Published:** 2015-09-22

**Authors:** Aiqin Liu, Jia Zhang, Jingmin Zhao, Wei Zhao, Rongjun Wang, Longxian Zhang

**Affiliations:** Department of Parasitology, Harbin Medical University, Harbin, Heilongjiang 150081 China; The Turbine Hospital of Harbin, Harbin, Heilongjiang 150040 China; College of Animal Science and Veterinary Medicine, Henan Agricultural University, Zhengzhou, Henan 450002 China

**Keywords:** *Cryptosporidium andersoni*, Multilocus sequence typing, Subtypes, Horses, Diarrhea

## Abstract

**Background:**

Horses interact with humans in a wide variety of sport competitions and non-competitive recreational pursuits as well as in working activities. *Cryptosporidium* spp are one of the most important zoonotic pathogens causing diarrhea of humans and animals. The reports of *Cryptosporidium* in horses and the findings of zoonotic *Cryptosporidium* species/genotypes show a necessity to carry out molecular identification of *Cryptosporidium* in horses, especially in diarrheic ones. The aim of the present study was to understand *Cryptosporidium* infection and species/genotypes in diarrheic horses, and to trace the source of infection of horse-derived *Cryptosporidium* isolates at a subtype level.

**Findings:**

Fecal specimens of 29 diarrheic adult horses were collected in Taikang County in northeastern China’s Heilongjiang Province. *Cryptosporidium* oocysts were concentrated by Sheather’s sugar flotation technique, and then examined by a bright-field microscope. Meanwhile, all the specimens were subjected to PCR amplification of the small subunit (SSU) rRNA gene of *Cryptosporidium. C. andersoni* isolates were further subtyped by multilocus sequence typing (MLST) at the four microsatellite/minisatellite loci (MS1, MS2, MS3 and MS16).

One and two *Cryptosporidium*-positive isolates were obtained in horses by microscopy and by PCR, respectively. The two *C. andersoni* isolates were identified by sequencing of the SSU rRNA gene of *Cryptosporidium.* Both of them were identical to each other at the MS1, MS2, MS3 and MS16 loci, and MLST subtype A4,A4,A4,A1 was found here.

**Conclusions:**

This is the first report of *C. andersoni* in horses. The fact that the MLST subtype A4,A4,A4,A1 was reported in cattle suggests a large possibility of transmission of *C. andersoni* between cattle and horses.

## Findings

### Background

*Cryptosporidium* spp. is a common zoonotic enteric pathogen responsible for diarrheal diseases in humans and a variety of animals worldwide. The diarrhea caused by cryptosporidiosis varies depending on the health status of the infected hosts. In people with various immune-system deficiencies, *Cryptosporidium* infection may continue and become life-threatening. Many studies have confirmed the high mortality associated with *Cryptosporidium* infection in HIV-infected patients [1]. Current PCR-based molecular techniques have made it possible to identify morphologically indistinguishable species/genotypes and subtypes, and give a more precise definition of host specificity, zoonotic potential, and transmission pathways of *Cryptosporidium* spp.. To date, at least 27 *Cryptosporidium* species and more than 40 genotypes have been recognized, with new genotypes being found [2, 3]. Most of them are host-adapted and have a narrow host range, such as *C. cani*s, *C. felis*, *C. muris*, *C. andersoni* and *C. suis* mainly in dogs, cats, rodents, cattle and pigs, respectively; in contrast, some species/genotypes, most notably *C. parvum* and *C. ubiquitum*, have a broader host range [2]. At present, due to the absence of effective vaccine and the lack of prophylactic and therapeutic drugs for cryptosporidiosis, understanding the molecular epidemiology and transmission dynamics of *Cryptosporidium* is an important step in controlling *Cryptosporidium* infection in humans and in avoiding outbreaks of cryptosporidiosis.

Horses are common animals worldwide, and can be used for leisure activities, sports, and working purposes. The findings of zoonotic pathogens in horses have highlighted that horse health is not only a veterinarian issue, but also a public health issue. Horse cryptosporidiosis was initially described in immunodeficient Arabian foals with severe diarrhea [4]. This disease has also been reported in immunocompetent horses [5, 6]. However, epidemiological data of cryptosporidiosis, especially molecular data, are still scarcer in horses. In China, limited reports of horse cryptosporidiosis are currently available [7, 8]. In the present study, two *C. andersoni* isolates were identified in adult horses with diarrhea by PCR amplification and sequencing of the small subunit ribosomal RNA (SSU rRNA) gene of *Cryptosporidium*. Both of them were further subtyped by multilocus sequence typing (MLST) at the four microsatellite/minisatellite loci (MS1, MS2, MS3 and MS16). The infection source of horse-derived *C. andersoni* was evaluated based on subtyping results.

### Methods

#### Ethics statement

The present study was carried out strictly in accordance with the recommendations of the Guide for the Care and Use of Laboratory Animals of the Ministry of Health, China. The research protocol was reviewed and approved by the Research Ethical Committee of Harbin Medical University. Before beginning the present study, we contacted the owners or managers of these animals and obtained their permissions to have their animals involved. No specific permissions were required for the described field studies due to the fact that the field studies did not involve endangered or protected species. During collection of fecal specimens, no animals were harmed.

#### Collection of fecal specimens

29 diarrheic adult horses (aged 2.5 to 7 years) were selected from individual owners and all of them shared the same grassland locating in Taikang County of northeastern China’s Heilongjiang Province. One fecal specimen (10–15 g) of each animal was collected from the ground immediately after defecation by using a sterile disposable latex glove and was then placed into an individual sterile plastic container. All the specimens were transported to the laboratory within 24 h and stored in refrigerators at 4 °C. The selected animals here had no previous history of horse parasitization.

#### Specimen processing and detection of *Cryptosporidium* oocysts

To reduce interference resulting from crude fiber and impurities in horse manure in detection of *Cryptosporidium* oocysts, each fecal specimen was homogenized with distilled water, filtered with a sieve and centrifuged at 1500 g for 10 min. Approximately 2 g of each sediment was used to concentrate *Cryptosporidium* oocysts by Sheather’s sugar flotation technique and the concentrates were detected for the presence of *Cryptosporidium* oocysts by bright-field microscopy under × 400 and × 1000. The remaining sediment was stored in 2.5 % potassium dichromate at 4 °C prior to being used in molecular identification.

#### DNA extraction

Potassium dichromate was washed off with distilled water by centrifugation at 1500 g for 10 min four times at room temperature. Genomic DNA of *Cryptosporidium* was extracted from 200 mg of each of 29 stored fecal specimens using a commercially available kit (QIAamp DNA Mini Stool Kit, Qiagen, Hilden, Germany) according to the manufacturer-recommended procedures. Eluted DNA (200 μl) was kept frozen at −20 °C until its analysis with PCR.

#### Identification of *Cryptosporidium* spp./genotypes

An approximate 830 bp fragment of the SSU rRNA gene was amplified from all DNA preparations by a nested PCR using genus-specific primers of *Cryptosporidium* as previously described [9]. All the secondary PCR products positive for *Cryptosporidium* were sequenced and identified to *Cryptosporidium* species/genotypes.

#### MLST subtype of *C. andersoni*

Subtyping analysis for *C. andersoni* isolates were determined by amplifying the four minisatellite/microsatellite markers by nested PCRs, respectively, including MS1 coding for hypothetical protein, MS2 coding for 90 kDa heat shock protein, MS3 coding for hypothetical protein, and MS16 coding for leucine rich repeat family protein. The expected fragment lengths were approximately 550 bp, 450 bp, 530 bp and 590 bp, respectively, and primers and amplification conditions in PCR analysis were performed as described previously [10]. The secondary PCR products of each gene amplified were sequenced using the respective secondary primers.

##### Nucleotide sequence analysis

All positive secondary PCR products were directly sequenced with secondary PCR primers on an ABI PRISMTM 3730 DNA Analyzer (Applied Biosystems, USA), using a BigDye Terminator v3.1 Cycle Sequencing kit (Applied Biosystems, Foster, CA, USA). Accuracy of the sequencing data was confirmed by two-directional sequencing and a new PCR product if necessary. All the gene sequences obtained in the present study were aligned with each other and reference sequences obtained from GenBank by the Basic Local Alignment Search Tool (BLAST) (http://blast.ncbi.nlm.nih.gov/Blast.cgi) and Clustal X 1.83 (http://www.clustal.org/). *C. andersoni* subtypes were named according to the numbers in microsatellite/minisatellite repeats or/and nucleotide diversity in non-repeat regions at each locus [11].

### Results and discussion

Molecular data have confirmed the presence of eight *Cryptosporidium* species/genotypes in horses, including *C. parvum*, horse genotype, *C. erinacei* (previously described as hedgehog genotype), *C. muris*, *C. hominis*, *C. tyzzeri*, *C. felis* and *C. ubiquitum* [5–8, 12–24] (Table 1). In the present study, one and two positives of 29 fecal specimens were detected by microscopy after Sheather’s sugar flotation technique and by PCR amplification of the partial SSU rRNA gene of *Cryptosporidium*, respectively. The two *Cryptosporidium* isolates were both identified as *C. andersoni* by DNA sequencing. To the best of our knowledge, this is the first report of *C. andersoni* in horses.

**Table 1:** *Cryptosporidium* species/genotypes of natural infection identified in horses worldwide

Country	No. of isolates	*Cryptosporidium* species/genotypes (n)	Ref
Algeria	9	*C. parvum* (3); *C. erinacei* (4); *C. muris* (1); *C. hominis* (1)	[12, 13]
China	42	*C. parvum* (31); horse genotype (7); *C. felis* (2); *C. andersoni* (2)	[7, 8]; This study
Czech Republic	15	*C. parvum* (5); horse genotype(2); *C. muris* (7); *C. tyzzeri* (1)	[14–16]
Germany	1	*C. parvum* (1)	[7]
Italy^a^	72	*C. parvum* (31); horse genotype (41)	[18–21]
New Zealand	16	*C. parvum* (16)	[5, 6]
Poland	2	*C. muris* (2)	[16]
UK	3	*C. parvum* (2); *C. ubiquitum* (1)	[22, 23]
USA	9	horse genotype (9)	[24]
Total	169	*C. parvum* (89); horse genotype (59); *C. muris* (10); *C. erinacei* (4); *C. felis* (2); *C. andersoni* (2); *C. hominis* (1); *C. tyzzeri* (1); *C. ubiquitum* (1)	

^a^There are nine cases of mixed infection of *C. parvum* and horse genotype in Italy

*C. andersoni* is the predominant species responsible for bovine cryptosporidiosis and is mostly identified in asymptomatic juvenile and mature cattle. To elucidate the source attribution of infection/contamination of *C. andersoni* in horses, the two *C. andersoni* isolates were amplified by nested PCRs at the four minisatellite/microsatellite loci, MS1, MS2, MS3 and MS16. Both of them were identified as haplotypes A4, A4, A4, and A1 at the four loci, respectively. This MLST subtype has been reported to be the most common in dairy and beef cattle in Heilongjiang Province, in China [11]. The consistency of MLST subtype of *C. andersoni* isolates from horses and cattle suggested *C. andersoni* had a large possibility of circulation between cattle and horses. In the investigated area, cattle are one of main economic animals, and horses and other herbivorous animals, such as sheep and goats, often share the same pasture with large populations of cattle. This provides a feasible opportunity to transmit *C. andersoni* oocysts from cattle to horses, sheep and goats through contamination of the environment by animal feces. Indeed, MLST subtype A2,A4,A2,A1 of *C. andersoni* was identified in cattle and sheep in the same area [25]. Recently, MLST subtype A4,A4,A4,A1 of *C. andersoni* was found in another herbivorous animal, Bactrian camel in China [26]. Combined with the findings above, *C. andersoni* was inferred to possibly transmit from cattle to other herbivorous animals. Therefore, it is important to take measures to reduce/control occurrence of transmission of *C. andersoni* between these herbivorous animals via harmless disposal of animal manure while eliminating parasites.

### Conclusion

This is the first report of *C. andersoni* in horses, expanding the host range of *C. andersoni* and increasing the number of *Cryptosporidium* species/genotypes*.* Identification of MLST subtype A4,A4,A4,A1 of horse-derived *C. andersoni* suggests a possible transmission of *C. andersoni* between cattle and horses.

## References

[1] Mor SM, DeMaria A, Griffiths JK, Naumova EN (2009). Cryptosporidiosis in the elderly population of the United States. Clin Infect Dis.

[2] Ryan U, Fayer R, Xiao L (2014). *Cryptosporidium* species in humans and animals: current understanding and research needs. Parasitology.

[3] Ryan U, Paparini A, Tong K, Yang R, Gibson-Kueh S, O’Hara A, Lymbery A, Xiao L (2015). *Cryptosporidium huwi* n. sp. (Apicomplexa:Eimeriidae) from the guppy (*Poecilia reticulata*). Exp Parasitol.

[4] Snyder SP, England JJ, McChesney AE (1978). Cryptosporidiosis in immunodeficient Arabian foals. Vet Pathol.

[5] Grinberg A, Oliver L, Learmonth JJ, Leyland M, Roe W, Pomroy WE (2003). Identification of *Cryptosporidium parvum* ‘cattle’ genotype from a severe outbreak of neonatal foal diarrhoea. Vet Rec.

[6] Grinberg A, Pomroy WE, Carslake HB, Shi Y, Gibson IR, Drayton BM (2009). A study of neonatal cryptosporidiosis of foals in New Zealand. N Z Vet J.

[7] Guo PF, Chen TT, Tsaihong JC, Ho GD, Cheng PC, Tseng YC, Peng SY (2014). Prevalence and species identification of *Cryptosporidium* from fecal samples of horses in Taiwan. Southeast Asian J Trop Med Public Health.

[8] Qi M, Zhou H, Wang H, Wang R, Xiao L, Arrowood MJ, Li J, Zhang L (2015). Molecular identification of *Cryptosporidium* spp. and *Giardia duodenalis* in grazing horses from Xinjiang, China. Vet Parasitol.

[9] Xiao L, Singh A, Limor J, Graczyk TK, Gradus S, Lal A (2001). Molecular characterization of *cryptosporidium* oocysts in samples of raw surface water and wastewater. Appl Environ Microbiol.

[10] Feng Y, Yang W, Ryan U, Zhang L, Kvác M, Koudela B, Modry D, Li N, Fayer R, Xiao L (2011). Development of a multilocus sequence tool for typing *Cryptosporidium muris* and *Cryptosporidium andersoni*. J Clin Microbiol.

[11] Zhao W, Wang R, Zhang W, Liu A, Cao J, Shen Y, Yang F, Zhang L (2014). MLST subtypes and population genetic structure of *Cryptosporidium andersoni* from dairy cattle and beef cattle in northeastern China’s Heilongjiang Province. PLoS One.

[12] Laatamna AE, Wagnerová P, Sak B, Květoňová D, Aissi M, Rost M, Kváč M (2013). Equine cryptosporidial infection associated with *Cryptosporidium* hedgehog genotype in Algeria. Vet Parasitol.

[13] Laatamna AE, Wagnerová P, Sak B, Květoňová D, Xiao L, Rost M, McEvoy J, Saadi AR, Aissi M, Kváč M (2015). Microsporidia and *Cryptosporidium* in horses and donkeys in Algeria: detection of a novel *Cryptosporidium hominis* subtype family (Ik) in a horse. Vet Parasitol.

[14] Hajdusek O, Ditrich O, Slapeta J (2004). Molecular identification of *Cryptosporidium* spp. in animal and human hosts from the Czech Republic. Vet Parasitol.

[15] Ryan U, Xiao L, Read C, Zhou L, Lal AA, Pavlasek I (2003). Identification of novel *Cryptosporidium* genotypes from the Czech Republic. Appl Environ Microbiol.

[16] Wagnerová P, Sak B, McEvoy J, Rost M, Perec Matysiak A, Ježková J, Kváč M (2015). Genetic diversity of *Cryptosporidium* spp. including novel identification of the *Cryptosporidium muris* and *Cryptosporidium tyzzeri* in horses in the Czech Republic and Poland. Parasitol Res.

[17] Imhasly A, Frey CF, Mathis A, Straub R, Gerber V (2009). *Cryptosporidiose* (*C. parvum*) in a foal with diarrhea. Schweiz Arch Tierheilkd.

[18] Caffara M, Piva S, Pallaver F, Iacono E, Galuppi R (2013). Molecular characterization of *Cryptosporidium* spp. from foals in Italy. Vet J.

[19] Perrucci S, Buggiani C, Sgorbini M, Cerchiai I, Otranto D, Traversa D (2011). *Cryptosporidium parvum* infection in a mare and her foal with foal heat diarrhoea. Vet Parasitol.

[20] Veronesi F, Passamonti F, Cacciò S, Diaferia M, Piergili Fioretti D (2010). Epidemiological survey on equine *cryptosporidium* and *giardia* infections in Italy and molecular characterization of isolates. Zoonoses Public Health.

[21] Galuppi R, Piva S, Castagnetti C, Iacono E, Tanel S, Pallaver F, Fioravanti ML, Zanoni RG, Tampieri MP, Caffara M (2015). Epidemiological survey on *Cryptosporidium* in an Equine Perinatology Unit. Vet Parasitol.

[22] Chalmers RM, Thomas AL, Butler BA, Morel MC (2005). Identification of *Cryptosporidium parvum* genotype 2 in domestic horses. Vet Rec.

[23] Li N, Xiao L, Alderisio K, Elwin K, Cebelinski E, Chalmers R, Santin M, Fayer R, Kvac M, Ryan U, Sak B, Stanko M, Guo Y, Wang L, Zhang L, Cai J, Roellig D, Feng Y (2014). Subtyping *Cryptosporidium ubiquitum*, a zoonotic pathogen emerging in humans. Emerg Infect Dis.

[24] Burton AJ, Nydam DV, Dearen TK, Mitchell K, Bowman DD, Xiao L (2010). The prevalence of *Cryptosporidium*, and identification of the *Cryptosporidium* horse genotype in foals in New York State. Vet Parasitol.

[25] Wang R, Jian F, Zhang L, Ning C, Liu A, Zhao J, Feng Y, Qi M, Wang H, Lv C, Zhao G, Xiao L (2012). Multilocus sequence subtyping and genetic structure of *Cryptosporidium muris* and *Cryptosporidium andersoni*. PLoS One.

[26] Liu X, Zhou X, Zhong Z, Deng J, Chen W, Cao S, Fu H, Zuo Z, Hu Y, Peng G (2014). Multilocus genotype and subtype analysis of *Cryptosporidium andersoni* derived from a Bactrian camel (*Camelus bactrianus*) in China. Parasitol Res.

